# Physiotherapists’ practice patterns for the diagnosis and management of patients with chronic contracted frozen shoulder in the United Arab Emirates

**DOI:** 10.1371/journal.pone.0283255

**Published:** 2023-03-24

**Authors:** Mariam J. Alhammadi, Fatma A. Hegazy

**Affiliations:** 1 Department of Physiotherapy, College of Health Sciences, University of Sharjah, Sharjah, United Arab Emirates; 2 Sharjah Social Services Department, Government of Sharjah, Sharjah, United Arab Emirates; 3 Faculty of Physical Therapy, Cairo University, Giza, Egypt; Mugla Sitki Kocman Universitesi, TURKEY

## Abstract

**Background:**

Adhesive capsulitis or contracted shoulder, known as frozen shoulder, is a persistent painful condition that may last for more than three months. It is a common disease-causing morbidity that causes pain and loss of shoulder range of motion. Physical therapy is advocated for the restoration of a pain-free state and normal use of the upper extremity, along with other interventions.

**Purpose:**

This study aimed to explore the level of current clinical practice for managing chronic contracted frozen shoulder (CCFS) among physiotherapy professionals in the United Arab Emirates (UAE) compared to well–established evidence-based practices, and to identify the most common therapy practiced in UAE to manage CCFS.

**Method:**

This study was based on a cross-sectional quantitative analysis using an adapted questionnaire. The main themes of questions were the presence of a special interest in CCFS, management options, symptoms, diagnosis, referral, and best physiotherapy intervention recommendations. The results were analyzed using simple descriptive analyses, such as frequency, mean, and percentage of total responses; additionally, thematic and content analyses were performed for open-ended questions.

**Results:**

Overall, 101 physiotherapy professionals participated in the survey: 62% female and 38% male; 59% were bachelors- and 36% masters-degree holders, respectively. In the closed ended question regarding the interest in CCFS, male physiotherapists (PTs) were more interested than females (82% vs. 68%). For the most common indication of CCFS, 76% of the participants selected “Limitation of movement" as the main indication. However, only 42% confirmed the presence of clinical protocols in their employment setting. In their opinion, the most effective therapies were patient education, superficial heat or cold, manual joint mobilization, and sustained stretching exercises.

**Conclusion:**

A well-established professional competence exists among physiotherapists in the UAE to manage and treat patients with CCFS. The findings showed sufficient standard, theoretical, and practical knowledge among the study groups.

## Introduction

### Background

Adhesive Capsulitis or contracted Shoulder, also known as frozen shoulder, is a persistent painful condition that may last for more than three months. It is a common disease-causing morbidity that causes pain and loss of range of motion in the shoulder [1]. Although it is self-limited, it can persist for years and may never resolve. It is a common condition; however, its treatment and definition remain challenging [2, 3]. The natural history of adhesive capsulitis is not entirely understood; however, most patients experience the following stages of the condition: (a) a freezing or painful stage, (b) followed by stiffness, frozen, or a transitional phase, and (c) a thawing phase, characterized by an increased range of motion (ROM). The treatment options vary from rehabilitation as the initial conservative measure, anti-inflammatory drugs, intra-articular corticosteroids, and capsular distension injections, to surgical interventions in refractory cases [1].

A lack of consensus regarding the etiology of frozen shoulder, and subsequently whether to define it as an inflammatory condition or fibrosis, is observed in the literature. A few researchers have attempted to categorize patients into two subgroups to simplify the treatment options: (a) idiopathic/primary chronic contracted frozen shoulder (CCFS) when patients display symptoms with no identifiable cause and (b) secondary CCFS when patients had a similar presentation and progression from a known intrinsic, extrinsic, or systemic cause [1].

The main aim of the physical management of the condition is to restore the joint’s motion while minimizing shoulder pain. The manual treatments consist of manipulation and mobilization techniques to restore the range of a pain-free state and regular use of the upper extremities [4]. In addition, physiotherapists (PTs) should emphasize on patient education and adherence to follow appropriate daily home exercises to restore their active range of motion [5, 6].

The basic strategy in treating structural stiffness according to the stage of FS is to apply appropriate tissue physical stress, which is based on patient’s irritability classification, pain and ROM tolerance [7]. At this point, physiotherapy professionals need to consider the clinical presentation of the patient’s condition and the stage of frozen shoulder. Physiotherapy professionals will consider three main factors when calculating the dose or the total amount of stress delivered to a tissue, namely (a) intensity, (b) frequency, and (c) duration [8, 9].

The prevalence of frozen shoulder is estimated to be in 2–5% of the population and affects women more than men. They are primarily observed in patients aged 40–60 years [1, 10, 11]. Additionally, this condition exists in 10–38% of the population diagnosed with diabetes and thyroid diseases [4, 12, 13]. The significant impact of CCFS has been previously thought to heal naturally within one to two years after occurrence. However, studies showed that disability and normal functionality of the affected shoulder persists without adequate treatment [14]. The compromised shoulder mobility causes difficulty and restriction in performing activities of daily living (ADL) [15]. Functional impairments caused by frozen shoulder conditions include limited reaching, especially during overhead and to-the-side movements, such as hanging clothes and fastening one’s seat belt [1].

Therefore, there is a massive variation in the definition, diagnosis, and management of this disorder. However, more insights and exploration are required at the local level in the United Arab Emirates (UAE) to establish a baseline assessment of CCFS among physiotherapy professionals.

The main objective of this thesis is to assess the current knowledge practice (preferred treatment modalities for CCFS) in the UAE among physiotherapy professionals working in different settings, such as inpatients, outpatients, and academic settings. This study aimed to answer the following research questions: (a) Is there a standardized protocol based on evidence-based practice for the management of CCFS among physiotherapy professionals in the UAE? (b) What is the common understanding and level of knowledge about CCFS among physiotherapy professionals in the UAE compared to the existing knowledge in the literature? (c) What is the most common therapy practiced in UAE to manage CCFS among physiotherapy professionals? Therefore, the researcher would like to explore the following objectives to answer these questions: (a) to analyze the level of current clinical practice for managing CCFS among physiotherapy professionals in the UAE compared to well–established evidence-based practices; (b) to assess the current knowledge regarding the management of CCFS among physiotherapy professionals in the UAE regarding diagnosis, assessment, and treatment modalities; and (c) to identify the most common therapy practiced in the UAE to manage CCFS among physiotherapy professionals.

## Material and methods

### Study design

This study was based on a retrospective, cross-sectional quantitative method using an adapted questionnaire from Hanchard et al. [16] in the United Kingdom. The sampling approach was purposive and logically assumed to be representative of the population based on the expert knowledge of the proper population. The study sample size was 101 physiotherapy professionals in the UAE.

### Participants

Professionals with the designations of “physiotherapist” and “physiotherapy technician” of healthcare workers who live in the UAE and work in different healthcare settings located in the UAE were invited to participate in this study. The list of names was obtained from different resources: (a) the researcher’s personal network, (b) academic alumni, (c) social media, such as professional physiotherapy Whatsapp groups, and (d) from the healthcare setting administration. The selection criterion was based on having a degree in physiotherapy or related fields with any level of experience in post-graduate certificates or degrees. A total of 101 participants were included in this study via an electronic survey. All the participants provided consent before participation.

### Sampling and administration of survey

The sampling method used was purposive sampling, also known as judgmental, selective, or subjective sampling. It is a form of non-probability sampling in which the researchers depend on their judgment in selecting the population to participate in their surveys. The main objective of a purposive sample is to have a sample that can be logically assumed to be representative of the population, based on the expert knowledge of the proper population [17]. In each setting or through a primary contact person, the researcher will send an email enclosing the survey link and a welcome message to encourage participation. The survey was completed by collecting the data through Google Forms in an Excel sheet format. Data collection was carried out over a period of four weeks, during November and December 2021.

The original survey aimed to gain insights into the diagnosis and management of contracted (frozen) shoulder (CFS) in a sample of UK physiotherapists, thereby supporting the development of evidence-based clinical guidelines. The reason for choosing the same tool was its reliability and ability to answer the research questions. Therefore, the primary source of information was collected from physiotherapy professionals residing in the UAE.

An online survey titled “Physiotherapists’ Practice Patterns for Diagnosis and Management of Patients with Chronic Contracted Frozen Shoulder in the United Arab Emirates” was used and modified to suit the current context of the UAE [S1 Questionnaire]. The modifications mainly included demographic data to avoid altering the reliability and validity of the survey. However, this was identified as a limitation of the study: the survey shall be restructured and modified to suit the current types of health care settings in the UAE, to add the nationality of participants as the UAE population is a multicultural society and PT professionals are available from all over the world. Permission to reuse the questions in the study was obtained by contacting the Copyright Clearance Center in Elsevier Academic Publishing Company, and approval was obtained (**License Number: 5302361500945**).

The modifications mainly included demographic data to avoid altering the reliability and validity of the survey. The main themes of the questions were as follows: the presence of a particular interest in CCFS, management options, symptoms, diagnosis, referral, and best physiotherapy intervention (PTI) recommendations from their perception. The newly built electronic survey (using Google form) consisted of three main sections: an introduction along with consent, demographic data, and the final section with 15 questions. The questions inquired about their workplace, competency in practicing acupuncture or injection therapy, ability to identify the patients with CFS, suitable treatment modalities, and referral options. The questions varied from open-ended to close-ended questions or multiple choices, with the flexibility of choosing more than one options or adding other recommendations not listed by the researcher.

### Data analysis

The analysis of the survey questions was based on a simple descriptive analysis using the frequency, mean, and percentage of the total responses. For the demographic data and closed-ended questions (age, setting, years of experience, sex, and degree), the frequency of responses was presented as percentage to explain the perception or given information varied among the different variables. Few numerical data, such as age and experience, were grouped into four to six groups, as the 101 respondents listed their actual age, which yielded a wide range of data. Conditional distribution was used to analyze the relationship between the participants’ inputs and their demographic data. For open-ended questions, thematic and content analyses were performed according to each question, and the relative frequency was present for the most common responses. The analysis was performed using the built-in formulas in Microsoft Office Excel. Similarly, statistical graphs and other illustrations have been produced.

### Ethics

The newly built & modified electronic survey (using Google form) consists of three main sections: an introduction and participation consent and contact information, a section for demographic data, and the final section with 15 questions. Ethical considerations underwent structured processes through the University of Sharjah Research and Ethical Approval Committee (REC) in UAE.

The principal investigator and co-investigator submitted a research request through a written proposal to the Research and Ethical Committee at the University of Sharjah. The principal investigator and co-investigator consented to preserve all the confidential data collected from the participants according to the REC guidelines. The research proposal was approved on June 21, 2021, with reference number (REC-21-06-09-02-S).

## Results

A total of 101 subjects participated in the survey; the majority of the participants were women (62%), and (38%) were men. Most of the participants (61%) were aged 25–36 years, with an average age of 33.8 years. Approximately 59% of the participants were bachelors degree holders, followed by 36% masters degree holders; however, out of 36 masters degree holders, only 22 participants were observed to have a masters in physiotherapy (PT), while the diploma holders constituted approximately 3%, and few participants held diplomas in modern sciences or modern orthopedics. Approximately 47% of the participants worked in a mixed setting (outpatient and inpatients’ units), compared to 6% in academia. Approximately one-third of the study population had work experience ranging from six to 11 years. The majority of PTs with different levels of education were interested in CCFS, as displayed in Table 1.

**Table 1:** General characteristics of the participants in the survey.

**Sex**		**(n = 101)**		**Number**	**Percentage (%)**
		**Female**		63	62.3%
		**Male**		38	37.6%
**Age (n = 101)**	**Number**	**Percentage (%)**	**Entry level PT degree**	**Number**	**Percentage (%)**
**19–24 years**	6	5.9	**Bachelor**	60	59.4
**25–30 years**	29	28.7	**Master**	36	35.6
**31–36 years**	33	32.6	**Diploma**	3	2.97
**37–42 years**	21	20.7	**Doctorate/ DPT**	2	1.98
**43–48 years**	8	7.9	**Total**	**101**	**100**
**49–54 years**	4	3.9			
**Post PT degree**	**Number**	**Percentage (%)**	**Placement employment in the last 12 months**	**Number**	**Percentage (%)**
**None**	70	69.3	**Inpatient and outpatient orthopedics**	47	46.5
**Master**	28	27.7	**Outpatient orthopedics**	27	26.7
**Diploma**	2	1.98	**Orthopedics**	17	16.8
**PhD**	1	0.99	**Academia**	6	5.9
**Total**	**101**	**100**	**Others**	4	3.9
			**Total**	**101**	**100**
**Clinical experience (Year)**			**Number**	**Percentage (%)**	
**0 to 5**			30	29.7	
**6 to 11**			37	36.6	
**12 to 17**			20	19.8	
**18 to 25**			13	12.8	
**More than 25**			1	0.99	
**Total**			**101**	**100**	

The responses from PTs showed that male PTs were more interested in CCFS than female PTs (82% compared to 68%). The majority of participants with different levels of education had an interest in CCFS. In addition, 47% of CCFS cases were managed at secondary care (consultant referrals), followed by self-GP referral (43%) (Table 2).

**Table 2:** Presence of special interest in chronic contracted (frozen) shoulder compared to the participants’ gender, degree, and employment setting.

	**No, not interested**	**Yes, interested**	**Total Sex**
**Sex**			
**Female**	**20 (31.75%)**	**43 (68.25%)**	**63**
**Male**	**7 (18.42%)**	**31 (81.58%)**	**38**
Total (presence of Interest)	**27 (26.73%)**	**74 (73.27%)**	**101**
**Entry-level PT degree**	**No, not interested**	**Yes, interested**	**Total degree**
**Bachelor**	14 (23.33%)	46 (76.67%)	**60**
**Diploma**	3 (100.00%)	**-**	**3**
**Doctorate/ DPT**	**-**	2 (100.00%)	**2**
**Master**	10 (27.78%)	26 (72.22%)	**36**
Total (presence of Interest)	27 (27%)	74 (73%)	**101**
**Employment Setting**	**No, not interested**	**Yes, interested**	**Total (n = 101)**
**Inpatient and outpatient orthopedics**	9 (19%)	38 (81%)	**47**
**Outpatient orthopedics**	10 (37%)	17 (63%)	**27**
**Orthopedics**	2 (12%)	15 (88%)	**17**
**Academia**	3 (50%)	3 (50%)	**6**
**Others**	3 (75%)	1(25%)	**4**
**Total**	27 (27%)	74 (73%)	**101**

The clinical diagnosis of frozen shoulder arrived at by a physician and is based on a thorough history and physical examination. The most commonly used criteria are pain and significant limitations of active and passive shoulder motion (7). The main aim of the physical management of the condition is to reconstruct the joint’s motion while minimizing shoulder pain.

**Fig 1:**
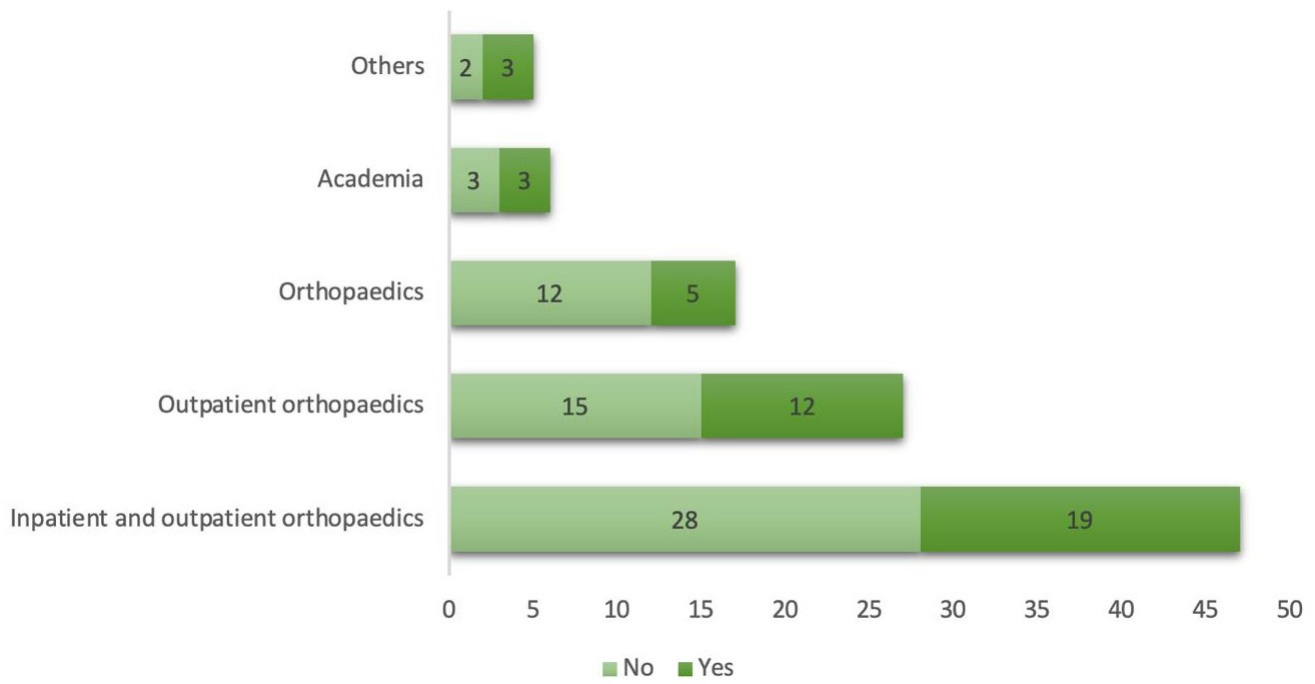
Presence of clinical protocol for the management of CCFS in different employment settings answered by physiotherapists in UAE, 2021.

Approximately 90% of bachelors holders preferred secondary referrals. The participants revealed that 42% had clinical protocols to manage CCFS in their employment setting, as shown in Fig 1. Notably, approximately half of the PTs did not practice acupuncture (54%), while the other half (46%) were practicing and working in inpatient and outpatient orthopedic settings.

When the participants were asked about considering imaging investigation, 26% of the participants agreed and 15% of the participants disagreed. Among these, 32% mentioned MRI as the recommended imaging investigation, followed by a combination of CT and MRI (9%), as shown in Table 3. Regarding the most common indication of CCFS, 76% of valid responses selected “Limitation of movement” as the main indication. This is followed by “Night pain or disturbed sleep.

**Table 3:** The participant’s agreement on the consideration, type, and reason of imaging investigation for CCFS.

**Agreement on the consideration and type of investigation**	
**MRI**	32 (31.6%)
**Yes**	26 (25.7%)
**No**	15 (14.8%)
**CT, MRI**	9 (8.9%)
Others	8 (7.9%)
**Radiography**	5 (4.9%)
**Radiography and MRI**	3 (2.9%)
**Diagnostic ultrasound**	3 (2.9%)
**Total**	101
**Reason of the imaging investigation for CCFS (91 responses), the most frequent six answers were taken**	
To identify or exclude bony abnormalities	30
To identify or exclude bony abnormalities; To investigate atypical presentations	9
To investigate unresponsive frozen shoulders	7
To identify or exclude bony abnormalities; To exclude the neck	6
To identify or exclude bony abnormalities; To investigate unresponsive frozen shoulders	6
To investigate atypical presentations	5

**Fig 2:**
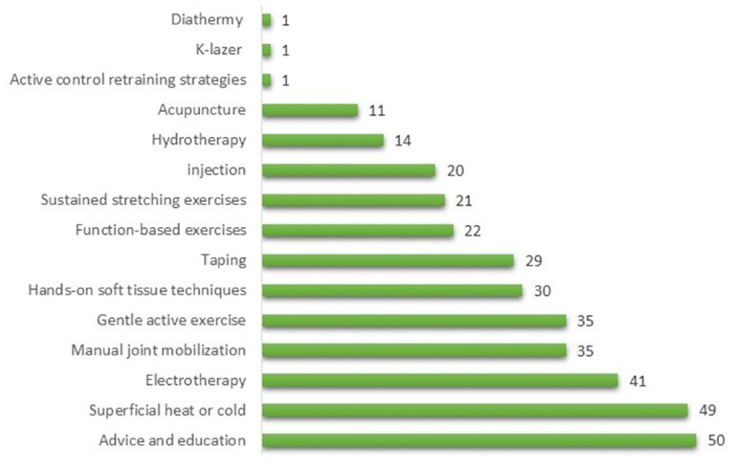
List of most frequent therapies for managing FS with the dominance of pain over stiffness by physiotherapists in UAE, 2021.

The seventh question in the survey had multiple inquiries related to the participants’ current competency and knowledge of the CCFS. For instance, the top five suggested therapies for managing CCFS with pain more than stiffness were (1) advice and education (mentioned 50 times); (2) superficial heat or cold (mentioned 49 times); (3) electrotherapy (mentioned 41 times); (4) manual joint mobilization (mentioned 35 times); and (5) gentle active exercise (mentioned 35 times), as shown in Fig 2.

**Fig 3:**
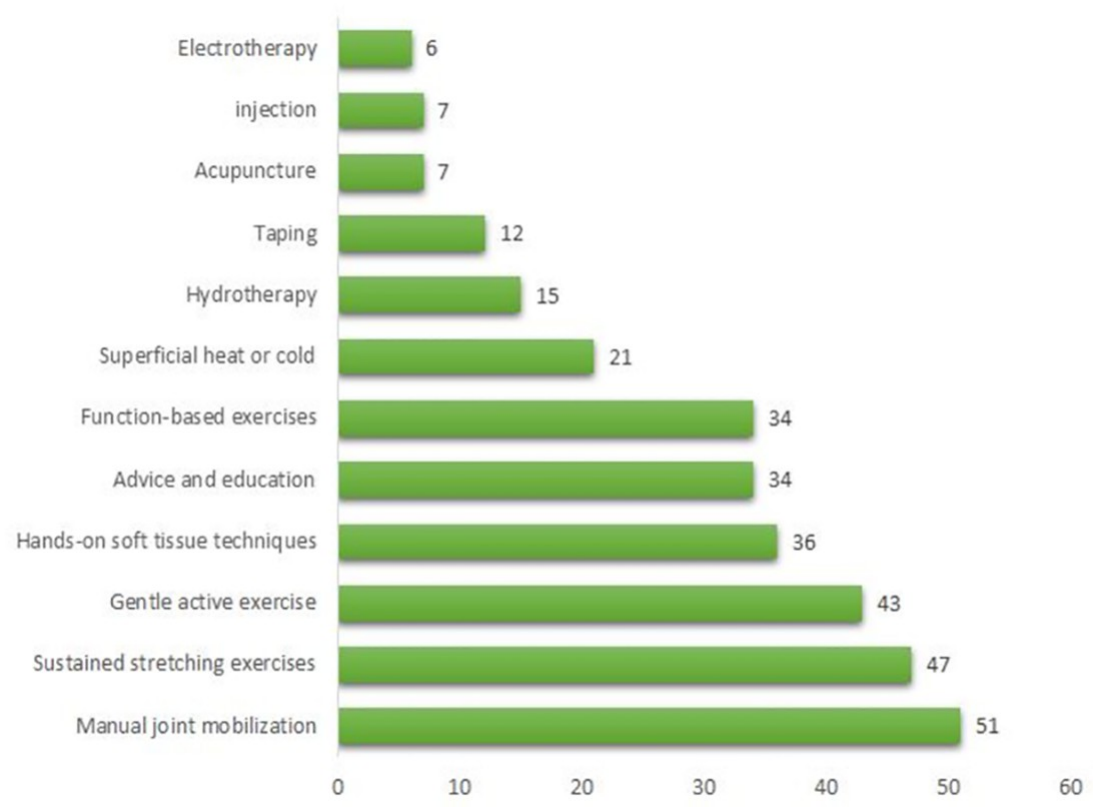
List of most frequent therapies for managing FS with the dominance of stiffness over pain by physiotherapists in UAE, 2021.

In contrast, the top five suggested therapies for managing CCFS with stiffness more than pain were: (1) manual joint mobilization (mentioned 51 times), (2) sustained stretching exercises (mentioned 47 times), (3) gentle active exercise (mentioned 43 times), (4) hands-on soft tissue techniques (mentioned 36 times), and (5) advice and education (mentioned 34 times), as shown in Fig 3.

## Discussion

The first objective was to explore the current level of clinical practice for managing CCFS among physiotherapy professions in the UAE. Based on these findings, approximately half of the studied population (PTs) preferred to send patients to secondary care or shoulder clinics. Raising a red flag to refer the patient to primary or secondary consultation is a required practice in physiotherapy, although it is not well established in the literature, as it mainly depends on the stage of CCFS. This is similar to a study in the UK, which tracked the patterns of referral of shoulder conditions in 2005; 22% of the patients were referred to secondary care up to three years following initial presentation, although most referrals occurred within three months [18]. Also, this finding aligns with the responses of PTs responses regarding the referral reasons, when 56% justified their referral to orthopedics for manipulation under anesthesia (MUA), followed by 41% for arthroscopic capsular release (ACR). However, only 42% of PTs stated that they had clinical protocols that described the management, pathways, or when to refer the patients based on these inputs. However, 58% remained without a written protocol, which is a gap that requires immediate attention for the sake of patient safety and the quality of clinical practice.

The second objective was to assess the current competencies in managing CCFS among physiotherapy professions in the UAE. As per the Emirates Physiotherapy Society (EPS), the scope of PT practice and competencies has been publicly published. These include specific practices with relevant continuing education like orthopedic manipulative therapy, acupuncture/meridian therapy, physiotherapy for older people, and others [19]. Additionally, in light of this study, there were two groups, namely 54% of the professionals who practice acupuncture, and 46% who do not practice acupuncture, although this practice is listed as part of the EPS scope and standard of care. However, the management of CCFS through acupuncture might not be within the scope of employment settings, and add to that; it requires additional intense training and evaluation.

Another area for assessing the competencies of PT in radiographic investigation, the majority (85%) agreed on such a process. Primary frozen shoulder is essentially a clinical diagnosis, and radiographic studies are therefore performed to exclude other secondary causes of shoulder pain, such as rotator cuff tear, calcific tendinitis, arthritis of the glenohumeral and acromioclavicular joint, or a neoplastic process [10]. However, one-third of PTs in this study suggested MRI as the primary radiographic investigation; nevertheless, MRI is not routinely performed in patients with CCFS to diagnose the condition [10]. Instead, it could be performed to rule out any secondary cause of CCFS if there is clinical suspicion. This might help in the prognosis of the condition, as per the presented stage. Another positive outcome in this assessment is the participants’ agreement on the most common signs and symptoms of CCFS, which is a limitation of movement. The index shoulder examination revealed global restriction of both active and passive range of movements (ROM) at least in two planes, which is one of the critical findings to exclude other reasons. A loss of external rotation with the arm by the side of the chest is one of the earliest signs [20].

In the third objective, the investigators assessed the common practices used by physiotherapists to deal with CCFS. As the condition and management vary according to the presented stage of CCFS, most PTs selected manual joint mobilization, sustained stretching exercises, and superficial heat or cold as the best practices in case of pain or stiffness. The effectiveness of these treatment approaches was reported in a Cochrane review of 28 of 32 randomized controlled trials, which stressed that exercises and mobilization are practical for frozen shoulder; however, there is no evidence that physiotherapy alone is beneficial [21]. It has been suggested that physiotherapists use manual treatments because they entail manipulation and mobilization techniques that help restore the range of a pain-free state and regular use of the upper extremity [4]. Additionally, the importance of patient education and advice is cited as the best approaches that physiotherapists could adopt. It has been suggested that patient education should encourage physiotherapists to test their active range of motion, thereby adopting appropriate therapies, such as home exercises. The other suggested strategies include electrotherapy, exercises, and soft tissue massage, which are congruent with previous studies [5, 6, 22].

## Conclusion

There is a well-established professional competence among participants who have adopted the best available evidence to manage and treat patients with CCFS. Additionally, the lack of a written protocol in a healthcare setting is not acceptable, and this gap should be addressed at the governance level, like at EPS. The findings also showed sufficient theoretical and practical knowledge among the study groups. Their recommendations for combined management suggested their full awareness of the required physical therapy or other operative interventions as the presented stage of CCFS.

Finally, concerning the most common therapy practiced in UAE to manage CCFS among physiotherapy professionals, the most common non-operative and operative interventions to treat CCFS were manual joint mobilization, sustained stretching exercises, gentle active exercise, hands-on soft tissue techniques, advice and education, superficial heat or cold, electrotherapy, MUA, and ACR.

### Strength and limitations

The study demonstrated a well-established professional competence among participants who adopted the best available evidence to manage and treat patients with FS. In addition, there was sufficient standardized theoretical and practical knowledge among the studied groups. Their recommendations for combined management suggested their complete awareness of the required physical therapy and other operative interventions as the presented stage of FS. However, challenges exist that need to be addressed in future research and opportunities for improvement. For instance, the built of the survey should be restructured and modified to suit the current types of healthcare settings in the UAE, to add the nationality of participants as the UAE population is a multicultural society and PT professionals come from all over the world. Understanding the impact of such diversity on clinical practice and different science schools is of great interest. In addition, survey scaling and the type of questions should be considered and made more definitive to prevent mixed answers, leading to an improper analysis. Another limitation is the involvement of physiatrists and orthopedics as part of the clinical pathway to understand and explore the current practice in all dimensions.

### Recommendation and future direction

Based on the current findings, these recommendations are proposed:

1. To conduct more studies on the prevalence and management of CCFS cases in the UAE for policymakers, such as EPS, and emphasize the importance of unifying the current management of CCFS.
2. To conduct a randomized clinical trial for the most common therapies suggested and establish strong evidence in the UAE.
3. To engage patients with CCFS in developing unified guidelines and what best works for the management of CCFS.

## Supporting information

S1 Questionnaire (DOCX)
