# Supplementary material for: Physiotherapists’ practice patterns for the diagnosis and management of patients with chronic contracted frozen shoulder in the United Arab Emirates
Source: PLoS One. 2023 Mar 24;18(3):e0283255. doi: 10.1371/journal.pone.0283255 (PMC10038245; doi:10.1371/journal.pone.0283255)
Supplement: S1 Questionnaire — (DOCX) [file pone.0283255.s001.docx]

| **Physiotherapists’ Practice Patterns for Diagnosis and Management of Patients with Chronic Contracted Frozen Shoulder in United Arab Emirates** | | | | | | | | | | | | | |
| --- | --- | --- | --- | --- | --- | --- | --- | --- | --- | --- | --- | --- | --- |
| **Section I : Informed Consent** | | | | | | | | | | | | | |
| It is our pleasure to invite you to participate in this online study. This research study is being conducted by Master student from the University of Sharjah, College of Health Sciences, Department of Physiotherapy, to investigate about the assessment, diagnosis and management of Frozen Shoulder CFS in a sample of UAE physiotherapists, strengthening the development of evidence-based clinical guidelines.   - The survey should take approximately 15-20 minutes to complete. - Your participation is completely voluntary. - The data will be kept strictly confidential and for scientific research purposes only. - You have the right to refuse or to withdraw the survey at any point. - If you have questions at any time about the survey or the procedures, you can contact:   Dr. Fatma Hegazy (email address: fhegazy@sharjah.ac.ae)   - If you have concerns or complaints about this study, please write to the University of Sharjah Research Ethics Committee at rec@sharjah.ac.ae.   Thank you for being interested to take part in our survey! | | | | | | | | | | | | | |
| I have read the description above and I agree to participate in this research study | | | | | | | | | | | | | |
| Yes | | | | | | No | | | | | | | |
| **Section I: Survey Participant Demographics** | | | | | | | | | | | | | |
| This section of the survey contains seven demographic questions about you. It should not take longer than 2 or 3 minutes to complete. If, after clicking the next button at the end of this section, you return here, please review your answers below and correct all replies highlighted in red. | | | | | | | | | | | | | |
| 1. Name (Optional) | | | | | |  | | | | | | | |
| 2. How old are you? | | | | | |  | | | | | | | |
| 3. What is your gender? | | | | | | Male | | | | | | | Female |
| 4. What is your entry-level PT degree? (Your first degree in physiotherapy) | | | | | | | | | | | | | |
| Diploma | Bachelor | | | | Master | | | | | | DPT | | Other |
| 5. Do you have any post-professional academic degree (additional to your entry-level PT degree)? Check all that apply. | | | | | | | | | | | | | |
| None | Master of Art | | | | Doctor of Health Sciences PhD | | | | | | ScD or DSc | | EdD |
| Master’s in health sciences Post professional master in PT Transitional DPT | | | | | | | | | | | | | Other |
| 6. In the last 12 months, what was your employment setting? | | | | | | | | | | | | | |
| Outpatient orthopedics/inpatient | Orthopedics | | | Inpatient and outpatient orthopedics | | | | | | | Academia | | Others |
| 7. How many years of clinical experience do you have? (Years that you worked only in academia or only in administration do not count.) | | | | | | |  | | | | | | |
| **Section III: Survey of Physiotherapists on The Diagnosis & Management of Chronic Contracted (Frozen) Shoulder** | | | | | | | | | | | | | |
| This section of the survey contains ten questions regarding the diagnosis and management of contacted frozen shoulder. If, after clicking the SUBMIT button at the end of the survey, you return here, please review your answers below and correct all replies highlighted in red. | | | | | | | | | | | | | |
| Question 1. Do you have a special interest in chronic contracted (frozen) shoulder? | | | | | | | | | | | | | |
| Yes, interested | | | | | | | No, not interested | | | | | | |
| Question 2. In which settings do you manage chronic contracted (frozen) shoulders? | | | | | | | | | | | | | |
| Primary care | | Secondary care (self- and GP referrals) | | | | | | Secondary care (consultant referrals) | | | | | |
| Question 3. Do you practice acupuncture? | | | | | | | | | | | | | |
| Practicing acupuncture | | | | | | | Not practicing acupuncture | | | | | | |
| Question 4. Do you practice injection therapy? | | | | | | | | | | | | | |
| Yes | | | | | | | No | | | | | | |
| Question 5. What symptoms and signs (or combination of symptoms and signs), if any, do you consider diagnostic of chronic contracted (frozen) shoulder? | | | | | | | | | | | | | |
| Limitation of movement | Night pain or disturbed sleep | | | | | | Inability to lie on the affected side or pain when attempting to do so | | | | | Other | |
| Question 6. Is your management of chronic contracted (frozen) shoulder dictated by a protocol? | | | | | | | | | | | | | |
| Yes | | | | | | | No | | | | | | |
| Question 7(a). Which treatment(s) might you use/recommend for a patient with chronic contracted (frozen) shoulder whose main problem was pain more than stiffness? | | | | | | | | | | | | | |
| Advice and education | Injection | | | | | | Superficial heat or cold | | | | | Acupuncture | |
| Electrotherapy | Gentle active exercise | | | | | | Hands-on soft tissue techniques | | | | | Function-based exercises | |
| Manual joint mobilization | Hydrotherapy | | | | | | Taping | | | Other | | Sustained stretching exercises | |
| Question 7(b). Which treatment(s) might you use/recommend for a patient with chronic contracted (frozen) shoulder whose main problem was stiffness more than pain? | | | | | | | | | | | | | |
| Advice and education | Injection | | | Superficial heat or cold | | | | | | | Acupuncture | | Electrotherapy |
| Gentle active exercise | Hands-on soft tissue techniques | | | Function-based exercises | | | | | | | Manual joint mobilization | | Hydrotherapy |
| Taping | Other | | | | | | Sustained stretching exercises | | | | | | |
| Questions 7a and 7b: * If your choice is electrotherapy, specify the modalities that you would use? | | | | | | | | | | | | | |
| TENS | Shortwave diathermy | | | | | | Ultrasound | | | | | Interferential | |
| Laser | Combination therapy | | | | | | Other | | | | |  | |
| Questions 7a and 7b: *Specify to whom you would consider referring for an injection at any stage of chronic contracted (frozen) shoulder? | | | | | | | | | | | | | |
| By physiotherapist | | | By GP | | | | | | By consultant | | | | |
| Question 8. Might you consider using/recommending any other conservative treatment(s) for a patient with chronic contracted (frozen) shoulder whose main problem was (a) pain more than stiffness or (b) stiffness more than pain? | | | | | | | | | | | | | |
| Answer: | | | | | | | | | | | | | |
| Question 9. Would you ever consider requesting/suggesting imaging investigations for chronic contracted (frozen) shoulder? (If yes, specify which type of investigation you would request or suggest?) | | | | | | | | | | | | | |
| Answer: | | | | | | | | | | | | | |
| Question 9a why you suggesting imaging investigations for chronic contracted (frozen) shoulder ?, If you answered yes in Q9. | | | | | | | | | | | | | |
| To identify or exclude bony abnormalities | | | To investigate atypical presentations | | | | | | To investigate unresponsive frozen shoulders | | | | |
| To inform treatment planning | | | To exclude the neck | | | | | | Other | | | | |
| Question 10. Would you ever consider referring a patient with chronic contracted (frozen) shoulder for an orthopedic opinion? | | | | | | | | | | | | | |
| Yes | | | | | | | No | | | | | | |
| Question 10a If you answered yes, For which patients might be referred? | | | | | | | | | | | | | |
| Specified referral for manipulation under anesthesia | Specified referral for arthroscopic capsular release | | | | | | Specified referral for distension | | | | | Other | |
